# The Effectiveness of Home-Based Training Software Designed to Influence Strategic Navigation Preferences in Healthy Subjects

**DOI:** 10.3389/fnhum.2020.00076

**Published:** 2020-03-17

**Authors:** Milan N. A. van der Kuil, Andrea W. M. Evers, Johanna M. A. Visser-Meily, Ineke J. M. van der Ham

**Affiliations:** ^1^Department of Health, Medical and Neuropsychology, Leiden University, Leiden, Netherlands; ^2^Center of Excellence in Rehabilitation Medicine, Brain Center, University Medical Center Utrecht, and De Hoogstraat Rehabilitation, Utrecht, Netherlands; ^3^Department of Rehabilitation, Physical Therapy Science and Sports, Brain Center, University Medical Center Utrecht, Utrecht, Netherlands

**Keywords:** spatial navigation, navigation strategy, cognitive training, strategy training, serious game

## Abstract

One approach to the rehabilitation of navigation impairments is to train the use of compensatory egocentric or allocentric navigation strategies. Yet, it is unknown whether and to what degree training programs can influence strategic navigation preferences. In validating this approach, the key assumption that strategic preference can be changed by using a navigation training was assessed in a group of healthy participants (*n* = 82). The training program consisted of a psychoeducation session and a software package that included either allocentric or egocentric navigation exercises in virtual environments. Strategic navigation preference, objective and self-reported spatial abilities were assessed in pre- and post-training sessions. Based on their pre-training strategic preference, participants received either the egocentric training (*n* = 19) or the allocentric training (*n* = 21) version of the training. These participants engaged in four training sessions over a period of 2–3 weeks. The second group of participants did not use the training software (*n* = 43) and served as a control group. The results show that 50% of participants that received the egocentric training shifted from an allocentric to and an egocentric strategic preference. The proportion of participants that switched their strategic preference as a result of the allocentric training was identical to this proportion in the control group (19%). The training did not affect objective and self-reported navigation abilities as measured in the pre- and post-training sessions. We conclude that strategic navigation preferences can be influenced by using home-based training in healthy participants. However, using the current approach, only a preference shift from an allocentric to an egocentric navigation strategy could be achieved. The effectiveness of this navigation strategy training should next be assessed in relevant patient populations.

## Introduction

Spatial navigation is a complex cognitive ability that is essential to our daily functioning. On a daily basis, humans traverse a range of environments (e.g., a crowded city or an open rural environment), with different navigational goals (e.g., exploration, finding one’s way home). In order to adapt to the variety of spatial challenges we are faced with regularly, evolution favored a complex and flexible navigation system in the human brain (Cashdan and Gaulin, 2016). Neuroimaging and lesion studies have identified a large neural network associated with spatial navigation, including the hippocampal formation, parahippocampal gyrus, retrosplenial cortex, medial temporal lobe, prefrontal cortex, precuneus and regions of the parietal lobe (Maguire et al., 1999; Chrastil, 2013; Boccia et al., 2014; Spiers and Barry, 2015). This widespread recruitment of the brain renders the navigation ability highly vulnerable to brain damage. Disruption of neural networks involved in navigation often results in navigation impairments (also known as topological disorientation) as observed in patients with acquired brain injury (Claessen and van der Ham, 2017), neurodegenerative diseases (Kalová et al., 2005) and developmental (Lind et al., 2013) and mental disorders (Hanlon et al., 2006). Navigation impairments are known to have a debilitating effect on the daily life activities of patients (Aguirre and D’Esposito, 1999). As such, navigation impairments have been associated with lowered quality of life, heightened levels of spatial anxiety and reduced autonomy (van der Ham et al., 2013).

Developing a standardized treatment for navigation impairments has proven to be a challenge due to the multifaceted nature of spatial navigation (Maguire et al., 1999; Wolbers and Hegarty, 2010; Claessen and van der Ham, 2017). Problems reported by navigation impaired patients are diverse and deficits are often specific. This is illustrated by a wealth of rapports of patients displaying specific spatial impairments: difficulty encoding novel landmarks (Herdman et al., 2015), recognizing famous landmarks (Rainville et al., 2005), understanding the order in which landmarks are encountered (van der Ham et al., 2010), remembering what actions to take at a landmark to follow a route (van der Ham et al., 2010), utilizing maps (Suzuki et al., 1998), forming a topological understanding of an environment (Ino et al., 2007) or switching between spatial reference frames (Ruggiero et al., 2014).

Over the past years, training programs have been developed with the goal of improving navigation ability in healthy subjects and patients. Most training programs for healthy subjects have been directed towards knowledge acquisition of specific environments. Examples of these include training for firefighters (Bliss et al., 1997), evacuation scenarios (Burigat and Chittaro, 2016) and astronauts learning to orient themselves in a space station (Aoki et al., 2008). One notable training program that has been developed for healthy participants has been reported in a study in which pre-school children were trained for 12 weeks to enhance their spatial orientation skills. After engaging in a variety of spatial exercises, children were able to encode and utilize map-like knowledge of an environment, a spatial skill that normally arises years later in development (Boccia et al., 2014). Several training programs have been reported that were specifically tailored to the impairments of a patient (Brooks, 1999; Incoccia et al., 2009; Bouwmeester et al., 2015; Claessen et al., 2016a). Some rehabilitation programs have focused on learning how to navigate a specific route through the environment (errorless learning; Lloyd et al., 2009) while other programs aimed to strengthen general spatial abilities by developing route learning (Kober et al., 2013). Generally, patients do benefit from navigation rehabilitation training. However, previous training programs have been either specifically designed for an individual patient or were directed at training navigation in a specific, spatially limited environment. Furthermore, the programs involve intensive supervision of experts as training programs required repeated sessions.

There is a need for a standardized navigation training that can be used to treat a broad range of navigation impairments. To account for the diversity in navigation impairments, the training should include exercises for navigational abilities in different spatial domains. Becoming acquainted with different navigation abilities should allow for the development of a more beneficial, compensatory navigation strategy, which can be used in real life. In order for this standardized training to be feasible in today’s healthcare system, the training should include both face-to-face therapy and repeated (unsupervised) training sessions (Wentzel et al., 2016). To this end, we propose a home-based navigation rehabilitation training that can be installed on and used from a patient’s home computer. Training exercises provided by the software should be modeled after experimental paradigms described in the field of spatial cognition.

When interacting with an environment, humans encode, update and process spatial information using distinct representations of space, referred to as reference frames (Klatzky, 1998). Spatial information about objects in the environment, in relation to the navigator’s own body is encoded into a body-centered, egocentric reference frame. Spatial relations between objects in the environment, irrespective of the navigators own position, are encoded into a world-centered, allocentric reference frame. The type of spatial information that is encoded and used during navigating reflects the employed navigation strategy. Remembering sequences of bodily turns (Iglói et al., 2009), landmark-direction associations at intersections (Wiener et al., 2013) and path integration (Wang et al., 2006) are all spatial abilities that rely on egocentric reference frames. As such, spatial behavior that relies on these abilities can be regarded as an egocentric navigation strategy. Conversely, spatial abilities such as place finding (Parslow et al., 2004), utilizing configurational knowledge of landmarks (Iglói et al., 2009) and the use of maps during navigation (Palermo et al., 2012), makes use of a world-oriented, allocentric reference frame. Spatial behavior that focusses on external cues during navigation can be classified as an allocentric navigation strategy. It is well established that (partially) distinct neural subsystems underlie navigation based on egocentric and allocentric reference frames (Jordan et al., 2004; Zaehle et al., 2007; Boccia et al., 2014; Colombo et al., 2017). This distinction between navigation strategies and their underlying neural correlates, suggests that a compensatory rehabilitation approach might be an effective approach to rehabilitation of navigation impaired patients.

Compensatory and metacognitive strategy training programs are practice standards in the rehabilitation of cognitive functions after brain injury (Cicerone et al., 2000, 2005, 2011, 2019). Such training programs start with the construction of a strengths and weaknesses profile in which a patient’s impairments and intact cognitive abilities determined. Then, training is constructed that focusses on the improvement of the intact abilities and the development of strategies that are beneficial to a patient. In terms of navigation impairment, participants with intact egocentric abilities, but difficulties in the allocentric domain, should be trained to adopt an egocentric navigation strategy and vice versa.

It is currently unknown whether navigational strategies can be influenced by training interventions. The aim of the current study was to test the key assumption that strategic navigation preference can be influenced by using home-based navigation training. By validating the concepts of the training in healthy subjects, we will provide the basis for a randomized control trial with navigation impaired acquired brain injury patients. To demonstrate a change in strategic navigation preference, we will train participants to adopt a navigation strategy other than their naive strategic preference. To this end, a home-based navigation training was developed in the form of a serious game. Two versions of the game were constructed: a version designed to train allocentric navigation strategies and a version designed to train egocentric navigation strategies. In order to provide evidence that strategic shifts were the result of the training intervention, a control group was used that did not receive the intervention. In addition, we aim to provide insight into the mechanisms by which a shift in strategic preference might occur. We will explore to what degree individual differences in objective and subjective navigation abilities determine naive strategic preference. Furthermore, we aim to examine individual characteristics that could potentially predict training success.

We hypothesized that participants who used the training program would display a preference for the navigation strategy trained in a situation where using both strategies can be deployed. As we expected the training to induce the strategic preference shifts, we expected a higher proportion of strategy shifts in the training group compared to the control group. Second, we hypothesized that using the training will lead to increased performance on spatial abilities associated with the trained domain. Specifically, egocentric spatial abilities (e.g., route continuation) will improve after the egocentric training, and allocentric spatial abilities (e.g., location on map) will improve after allocentric training. No performance changes were expected in the control group. Third, we hypothesized that subjective navigation ability will increase after using the training, whereas no change in subjective navigation ability was expected in the control group.

## Materials and Methods

### Design

A pre-test–post-test design was employed in this study including a control group, consisting of a “control” and “control + psychoeducation” subgroup and an experimental group consisting out of an “allocentric training” subgroup and an “egocentric training” subgroup. Measurements took place during two sessions: pre- and post-training. These measuring phases were separated by a 2 week intervention period. During the pre-training session, participants completed the screening/general questionnaire, the strategy assessment task, the Virtual Tübingen testing battery, which measured objective navigational ability, wayfinding questionnaire, which measured self-reported navigation and four neuropsychological assessments. During the post-training session, participants again completed the strategy assessment task, the Virtual Tübingen testing battery, and the wayfinding questionnaire. Participants in the experimental condition would engage in either the allocentric or egocentric training software in the period between pre- and post-training sessions.

### Participants

Participants were recruited from the university campus using posters, the university’s recruitment website, and social media. The inclusion and exclusion criteria for the study were: (1) between 18 and 35 years old; (2) Dutch-speaking; (3) access to personal computer and internet; (4) willingness and capability to complete the training program; and (5) no history of neurological or psychiatric disorders. All participants were required to sign an informed consent form in order to participate and were compensated for participation in participant hour credits or with a monetary reward of 6 € per hour. The study was performed in concordance with the Declaration of Helsinki (2013) and was approved by Leiden University’s local ethics committee for psychological research.

### Materials

#### Tasks

##### Screening/General Questionnaires

All participants completed a screening questionnaire in which they filled in demographic characteristics such as age, gender, handedness, level of education and gaming experience. Furthermore, screening information about psychiatric or neurological disorders was obtained.

##### Navigation Strategy Assessment

Strategic navigation preference was assessed during the pre- and post-training sessions using an adapted version of the Starmaze (Iglói et al., 2009). Two variants of the Starmaze were used: the original environment described by Iglói et al. (2009) and a mirrored environment. The Starmaze consisted out of five alleys that formed a pentagon and five alleys that radiated from this pentagon. The alleys were surrounded by a small wall that could not be traversed. Surrounding the environment were two distinct mountains, two distinct forests, and two radio towers, which were visible throughout the maze. Participants were instructed to explore the environment to find the goal location, which was located in one of the arms. Upon finding the goal location, the text “Bravo” would be displayed on-screen and the next trial was started. Over the course of the first five trials (training trials), participants would start in the same arm of the maze and learn to find the goal location. In the 6th trial (probe trial) participants started in a different arm of the maze. Participants could navigate using either the sequence of left-right turns that was learned during the training trials or by determining their location based on the configuration of landmarks in the environment. Participants utilizing the turn sequence approach would end in an alley that was different from the goal location in the training trials. Participants that utilized the configuration of cues would end in the original ending alley.

The ending location and the travel path measured in the probe trial were used to identify egocentric, allocentric or mixed navigation strategies. Participants who ended at the different goal location, and thus utilized a sequential egocentric navigation strategy, were classified as egocentric navigators. Participants who traveled directly (using the shortest route) to the original goal location, and thus utilized the configuration of landmarks to orient themselves, were classified as allocentric navigators. Participants that initially followed the turn sequence strategy, but changed direction and headed for the original goal location, were classified as mixed navigators.

##### Subjective Navigation Ability

Self-reported navigation ability was assessed during the pre- and post-training sessions using the Wayfinding Questionnaire (de Rooij et al., 2017). The Wayfinding Questionnaire contains 22 items in three subscales: navigation and orientation (11 items), distance estimation (three items) and spatial anxiety (eight items). All items were rated on a seven-point Likert scale.

##### Objective Navigation Ability

Objective navigation ability was assessed during the pre- and post-training sessions using an adapted version of the Virtual Tübingen testing battery (Van Veen et al., 1998; Claessen et al., 2016b). Four routes through the city were selected that were comparable in terms of distance and number of intersections. Participants watched a video of a route through a virtual replication of the city of Tübingen. Participants were instructed to memorize as much as possible about the spatial characteristics of the route and the environment. Afterward, participants completed 6 tasks in which navigation abilities were assessed.

Participants completed two variations of the task at each measuring phase. In the first variation of the tasks, participants saw the route from a first-person perspective. In the second variation, participants observed a red arrow icon moving along a route from a birds-eye view, the map perspective. The camera was placed at a height of 38 m and was focused on the red arrow. The camera did not rotate with the arrow and thus, was always aligned in the same direction.

After viewing the video, a Route Sequence task was conducted. Participants had to indicate what action was taken sequentially at each intersection point along the route. Options were left-turn, right-turn or straight. No images of the related decision points were shown. Numbers 1–8 were listed and participants selected the arrow icon indicating the response options for each number. Scoring was based on the number of correct responses. A participant’s score was the sum of correct responses (ranging from 1 to 8).

Then the Route Continuation task was performed. Participants were presented with eight images of the intersection points in random order. Participants had to indicate whether they turned left, right or went straight ahead at each decision point by pressing the arrow keys left, right or up arrow, respectively. Scoring was based on the number of correct responses. A participant’s score was the sum of correct responses (ranging from 1 to 8).

Participants then performed the Point to Start and Point to End tasks. Participants were shown eight scenes taken along the route in random order. Participants were asked to indicate where the start/end location of the route was using a rotational device. In the first-person perspective version, the rotational device was placed horizontally on the desk in front of the participants. Participants were asked to point from the perspective shown in the image. In the dynamic map perspective version, the rotational device was placed vertically on the desk next to the monitor. Participants had to indicate the start/end location on the map, relative to the red arrow icon the camera was following. Scoring was based on the mean pointing deviation angle for each trial, ranging from 0 to 180 degrees deviation.

In the Distance Comparison task, participants were shown a target image and two response images. In the first-person perspective version, the images corresponded to locations visited along the route. In the dynamic map perspective version, the images were landmarks encountered along the route. Participants had to indicate which of the two response locations was closest to the target location (direct path distance). A participant’s score was the sum of correct responses (ranging from 1 to 8).

Finally, participants performed the Locations on Map task. Participants were shown a schematic map of the city including icons indicating starting and ending locations. In the first-person perspective version, participants were shown images of eight locations along the route in random order. Participants had to indicate the correct location on the city map using the mouse. In the dynamic map perspective version, participants had to indicate where landmarks were located on the city map. Scoring was based on the amount of pixels deviation from the correct location.

##### Neuropsychological Assessment

Four neuropsychological tests were performed to assess general cognitive ability. The Corsi Block tapping tasks, both forward and backward, were used to asses visuospatial working memory (Kessels et al., 2000). The WAIS VI Digit span test, both forward and backward, was used to assess verbal working memory (Wechsler, 1987). A digital 46-item adaptation of the Mental Rotation test was used to assess object-based transformation ability (Shepard and Metzler, 1971; Vandenberg and Kuse, 1978). An adaptation of the 12-item Santa Barabara perspective-taking test was used to assess egocentric transformation ability (Hegarty and Waller, 2004).

### Training Intervention

The training intervention consisted of a short psychoeducation session and home-based navigation training software that was used over the course of 2–3 weeks.

#### Psychoeducation

The psychoeducation session took 20–30 min. The experimenter placed a document with illustrations on the table and read an educational text for the participants. After reading the text aloud, the experimenter discusses the illustrations on the document to clarify the content. The educational text addressed the following topics: the formation of egocentric and allocentric reference frames and the use of egocentric and allocentric navigation strategies. It was explained that people are capable of using both strategies and that certain strategies are more effective in specific situations. To verify whether participants understood the concepts, participants were asked to give examples of both egocentric and allocentric navigation strategies they have used. Participants were told that they would engage in a training program designed to train egocentric or allocentric navigation strategies. Importantly, participants were not informed about their performance or strategy preference in the Starmaze and Virtual Tübingen tasks.

#### Home-Based Training Software

Two versions of the training were constructed. Participants would receive either the egocentric navigation training or the allocentric navigation training. Each training consisted of 3 modules that were designed to train spatial abilities that are central to either an egocentric or allocentric navigation strategy. The egocentric training was composed of the modules: “landmark-action association,” “turn-sequence” and “egocentric updating.” The allocentric training was composed of the modules: “place-finding: distal landmarks,” “place-finding: local landmarks” and “effective map-use.” Each module resembled a simple game, set in the theme of ancient Greece. A comprehensive description of the training modules can be found in the Supplementary Material (Supplementary Figures S1–S10).

The navigation training software was installed on the participants’ home computer. Participants received a personal account, which allowed for data transfer with an online server. *Via* the server, progress during the training could be stored and tracked. Furthermore, training adherence was recorded by storing training time and the number of trials started and completed. Participants were instructed to engage in at least four separate training sessions, in which all three training modules should be used. Mails reminding the participant to train were automatically sent two times per week.

During a single training session, participants were instructed to perform at least one attempt to increase their level in all three training modules that were available to them. Each training module contained four difficulty blocks. Each difficulty block was composed of three levels of increasing difficulty levels. All participants started on difficulty block 1. When engaging in a training session, participants completed three levels within a difficulty block. If participants scored 75% or more of the points obtainable over the levels, participants would advance to a higher difficulty block. If participants failed to obtain 75% of the points, participants would remain on the same difficulty block. Depending on the participant’s skill level and progress, a training session was estimated to take 10–15 min.

### Procedure

All participants were invited to the laboratory at the Faculty of Social Science at the Leiden University, where participants read the information letter and signed the informed consent form in accordance with the Declaration of Helsinki (2013). Participants filled in the screening/general questionnaire followed by the Wayfinding Questionnaire and completed the Starmaze task.

Participant was assigned to the control or training condition based on participation order. The first half of the participants were assigned to the control groups. The second half of the participants were allocated to the training condition. Participants allocated to the training condition were assigned to the egocentric or allocentric training depending on the navigation strategy displayed in the Starmaze. Participants ending in the allocentric ending location, thus displaying a mixed or allocentric navigation strategy, received the egocentric training program. Participants ending in the egocentric ending location received the allocentric training program.

Following the Starmaze task, participants would complete the Virtual Tübingen testing battery. Route and order of the perspective (first-person or map perspective) were counterbalanced between conditions. A 10-min break was introduced following the Virtual Tübingen test. After the break, the four neuropsychological tests were completed.

For participants in the control condition, the first session ended here. Participants in the experimental condition would continue to receive psycho-education and were instructed on how to use the home-training software. During the training period, participants in the experimental condition would practice with the navigation training software during four occasions. During a training session, participants were instructed to perform all three training modules at least once. A periodically repeating mail was sent to the participants, reminding them to use the training application.

After 2 weeks, participants were invited back to the lab to perform the post-intervention measurement. The Starmaze, Virtual Tübingen and Wayfinding Questionnaire were conducted. The session ended with a debriefing.

### Analysis

#### Demographics, Neuropsychological and Visuospatial Measures

MANOVA analysis was performed to assess potential differences between participants in the conditions. Demographic, neuropsychological and visuospatial scores were compared between conditions.

#### Navigation Strategy

A Fishers’ exact test was used to compare the proportions of participants who changed strategy between the pre- and post-training sessions. To assess the effect of psychoeducation, the proportion of strategy shifts in the control conditions was analyzed. Then, proportional analysis was performed on the control condition and the egocentric and allocentric training conditions. In order to assess whether factors other than condition determined strategy change, the proportional analysis was performed for gender, gaming experience and education between strategy shifters and those who did not shift. Binary logistic regression was performed to investigate the relationship between training adherence and strategic shift.

#### Objective Navigation Ability

The effect of condition on performance in the Virtual Tübingen tasks was analyzed using a differences score analysis. A difference score was calculated for each navigation task by subtracting the pre-training score from the post-training score. A MANOVA was used to assess the effect of condition (control, egocentric training or allocentric training) on performance change. Three participants had an extreme score (*Z* > 3) on the map perspective point to start task and were removed from the analysis.

#### Subjective Navigation Ability

The effect of condition on self-reported navigational ability, measured using the Wayfinding questionnaire, was analyzed using a differences score analysis. A difference scores for each of the subscales (Spatial Anxiety, Navigation and Orientation and Distance estimation) was calculated by subtracting the pre-training score from the post-training score. A MANOVA was used to assess the effect of condition (control, egocentric training or allocentric training) on wayfinding questionnaire change scores.

#### Interaction Between Strategic Preference, Preference Shift, and Navigation Abilities

To explore the interaction between strategic navigation preference and navigation abilities, a MANOVA was conducted with strategic preference at T1 as between-subject factor (egocentric, allocentric or mixed strategy) and performance on egocentric (composite score of route sequence, route continuation and point to start) and allocentric (composite score of point to end, distance estimation and location on map) tasks as dependent variables. Separate composite scores were calculated for the egocentric and allocentric tasks for the first-person and map-perspective tasks. A similar analysis was conducted with the self-reported navigational scores (spatial anxiety, navigation and orientation, and distance estimation) as dependent variables.

MANOVAs were conducted to assess differences in objective and self-reported navigation abilities between participants that shifted their strategic preferences between T1 and T2 and participant that did not shift strategic preference.

A binary logistical regression was conducted to assess whether performance on objective egocentric (composite score of route sequence, route continuation and point to start) and allocentric (composite score of point to the end, distance estimation and location on map) predicted strategic preference shifts. A similar analysis was performed with self-reported navigational abilities (spatial anxiety, navigation and orientation and distance estimation) as predictors.

## Results

### Participants and Demographics

One-hundred and twenty-nine participants were recruited into the screening procedure. To maintain a gender balance in the egocentric training condition, the sessions of 29 females and one male were terminated during screening as they displayed an egocentric navigation strategy in the Starmaze, while this condition was already filled. Revealing a clear gender effect for strategy preference (22.97% females vs. 42.85% males displayed an allocentric navigation strategy during the first Starmaze task). Seven participants were screened on the basis of exclusion criteria as they reported psychological or neurological disorders, two participants did not perform the training at home (or trained for less than 5 min), three participants were lost to attrition, two participants were wrongly classified into the allocentric training condition. As a result, 82 participants successfully completed the experiment.

Participant characteristics for each condition are presented in Table 1. A MANOVA revealed that were no differences in scores on visuospatial and neuropsychological assessments between conditions, *F*_(12,148)_ = 0.40, *p* > 0.05; Wilk’s *Λ* = 0.94, partial η^2^ = 0.03, nor were there differences between age, education and gaming experience between conditions, *F*_(6,154)_ = 0.77, *p* > 0.05; Wilk’s *Λ* = 0.94, partial η^2^ = 0.03. Independent *t*-tests did reveal that training time significantly differed between the egocentric and allocentric strategy training groups, *t*_(37)_ = 4.05, *p* < 0.01, and the number of trials completed in the allocentric strategy training group was significantly higher than in the egocentric strategy training group, *t*_(37)_ = −7.21, *p* < 0.01.

**Table 1:** Overview of demographics data, neuropsychological scores and training adherence.

	Control (*n* = 43)	Experimental (*n* = 39)	
		Egocentric training (*n* = 18)	Allocentric training (*n* = 21)
**Demographics**			
Age in years, *M (SD)*	22.42 (2.85)	22.44 (3.11)	21.48 (2.14)
Gender, *% female*	62.79	55.56	57.14
Education, *M (SD)*^†^	6.77 (0.43)	6.80 (0.43)	6.76 (0.44)
Gaming experience, *M (SD)*^‡^	1.51 (0.94)	1.72 (1.18)	1.67 (1.02)
**Neuropsychological test scores at T1**			
Corsi block tapping task forward span, *M* (*SD*)	6.51 (0.94)	6.33 (1.03)	6.43 (0.87)
Corsi block tapping task forward product score, *M* (*SD*)	66.3 (20.16)	62.11 (20.91)	63.76 (16.68)
Corsi block tapping task backward span, *M* (*SD*)	6.74 (0.82)	6.61 (0.92)	6.52 (0.81)
Corsi block tapping task backward product score, *M* (*SD*)	71.14 (17.98)	69.11 (18.76)	66.86 (16.93)
Digit span forward span, *M* (*SD*)	6.14 (1.21)	6.78 (1.39)	6.38 (1.43)
Digit span forward product score, *M* (*SD*)	60.67 (24.19)	69.56 (25.67)	64.52 (30.44)
Digit span backward span, *M* (*SD*)	5.35 (1.15)	5.83 (1.15)	5.38 (1.12)
Digit span backward product score, *M* (*SD*)	52.98 (22.13)	57.89 (21.96)	53.04 (22.34)
Santa Barbara perspective taking test, deviation, *M* (*SD*)	14.99 (9.15)	15.04 (9.05)	16.88 (9.48)
Mental rotation slope, accuracy, *M (SD*)	76.98 (12.09)	76.67 (12.97)	75.29 (11.93)
Mental rotation slope, reaction time, *M (SD*)	4,992.63 (2,822.06)	5,520.32 (2,047.83)	5,047.36 (2,765.06)
Mental rotation slope, ms/degree, *M (SD*)	19.11 (11.69)	24.06 (19.65)	20.2 (15.11)
**Training adherence**			
Training time in minutes, *M* (*SD*)	-	62.31 (31.95)*	30.70 (15.00)*
Training Trials completes, *M (SD)*	-	27.94 (8.29)*	78.90 (28.94)*

^†^Level of Education measured on the Verhage scale, a Dutch scale of education level ranging from 1 (low) to 7 (high; Verhage, 1964). ^‡^Gaming experience was measured on a five point scale, represented indicating *1* = 0–2 h/week, *2* = 2–4 h/week, *3* = 4–8 h/week, *4* = 8–12 h/week, *5* = 12+ h/week. *T-tests indicate significant differences between groups (*p* < 0.05).

### Strategy Change

**Figure 1:**
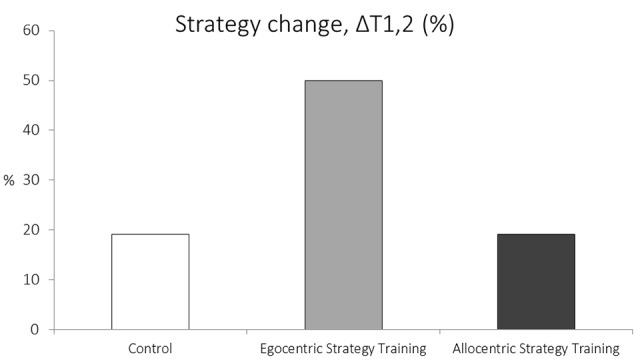
Proportion of participants that changed navigation strategy between the pre- and post-training sessions.

A Fisher’s Exact test revealed a significant effect of condition on the proportion of strategic preference changers (*p* < 0.05; *FET*, Figure 1). *Post hoc* analysis, using Bonferroni corrected Chi-squared tests, revealed that a higher proportion of participants changed strategy in the egocentric training condition compared to the control condition^1^ (50% vs. 19%), $\chi_{\left(1\right)}^{2}$ = 5.95, *p* = 0.015. *Post hoc* analysis did not reveal a significant difference between the proportion of participants that changed strategic preference after the “egocentric training condition” compared to the proportion of participants that changed strategic preference after the “allocentric training condition” (50% vs. 19%), $\chi_{\left(1\right)}^{2}$ = 4.18, *p* = 0.041 (not passing the Bonferonni correction). No significant differences were found between the allocentric training condition and the control condition in the proportion of participants that changed strategic preference (19% vs. 19%), $\chi_{\left(1\right)}^{2}$ = 0.0 *p* = 1. Overall, this analysis revealed that strategic preference shifts between pre- and post-training were present in all groups. However, the proportion of the participants who shifted strategic preference after receiving the egocentric training was significantly larger compared to the control group.

Additional proportional analyses were performed to determine whether strategic preference change could be attributed to other factors that are known to influence navigation strategy or learning processes. No effect of gender, $\chi_{\left(1\right)}^{2}$ = 0.65, *p* > 0.05, education, *p* > 0.05; *FET*, or gaming experience, *p* > 0.05; *FET*, was found. Training time and number of trials completed differed significantly between the egocentric and allocentric training groups (Table 1). Exploratory binary logistical regression analyses were conducted to explore whether strategy change could be attributed to these differences. Binary logistic regression revealed that there was no effect of training time on strategy change, $\chi_{\left(1\right)}^{2}$ = 1.07, *p* = 0.74. However, a significant relationship between the number of trials completed and strategy change was found, $\chi_{\left(1\right)}^{2}$ = 4.8, *p* < 0.028), with fewer trials completed leading to higher training success.

Inspection of the strategic preference changes shows that the direction of the change in the control condition was not uniform. Participants in the control group changed from egocentric to allocentric strategic preference and vice versa (Table 2).

**Table 2:** Direction of change in participant that changed navigation strategies between the pre- and post-training sessions.

Strategy T1	Strategy T2	Control (*n* = 42)	Egocentric training (*n* = 18)	Allocentric training (*n* = 21)
Egocentric	Allocentric	0	-	2
Egocentric	Mixed	1	-	2
Allocentric	Egocentric	2	5	-
Allocentric	Mixed	1	1	-
Mixed	Egocentric	3	2	-
Mixed	Allocentric	1	1	-

*The values in the table indicate the number of participants that changed strategic preference from T1 to T2. Participants that received the allocentric training, always displayed an egocentric strategy at T1 and could only shift towards a mixed or allocentric strategy. Vice versa, participants that received the egocentric training, always displayed a mixed or allocentric strategy at T1 and could shift towards any other strategy, not displayed at T1*.

### Objective Navigation Ability Assessment

MANOVAs were performed to test the hypothesis that navigation training leads to an increase in performance on the objective navigation tasks compared to the control group. Specifically, we expected that participants in the egocentric training condition had a higher, positive differences score on egocentric navigation tasks (route sequence, route continuation, point to start), whereas allocentric training would lead to higher, positive differences scores on allocentric navigation tasks (distance comparison, location on map, point to end). First, the analysis was run for the dynamic map perspective condition. A MANOVA on the difference scores (post-training—pre-training) of six navigation tasks as independent variables and conditions as a between-subject factor was performed (Table 3). A trend effect of condition was found on the differences scores *F*_(12,140)_ = 1.65, *p* = 0.07; Wilk’s *Λ* = 0.77, partial *η*^2^ = 0.13. Second, the analysis was run for the first-person learning condition. A MANOVA with the difference scores of six navigation tasks as independent variables and conditions as a between-subject factor was performed. No significant effect of condition was found on the differences scores *F*_(12,148)_ = 2.083, *p* > 0.05; Wilk’s *Λ* = 0.94, partial η^2^ = 0.03.

**Table 3:** Mean and standard deviation of performance on navigation tasks on the pre- and post-training sessions and differences scores.

	Control			Egocentric training			Allocentric training		
Subtask in virtual Tübingen battery	T1	T2	ΔT1, 2	T1	T2	ΔT1, 2	T1	T2	ΔT1, 2
**Map Perspective**								
Route sequence, % correct	61.01 (25.78)	70.35 (22.50)	8.93 (28.19)	76.39 (25.69)	61.81 (31.35)	−14.58 (38.41)	67.26 (23.54)	68.45 (25.50)	1.19 (36.64)
Route continuation, % correct	82.56 (17.71)	84.01 (14.52)	1.45 (19.33)	88.89 (9.48)	82.64 (16.12)	−6.25 (20.22)	79.17 (17.38)	80.95 (18.38)	1.79 (24.78)
Distance estimation, % correct	63.08 (15.66)	70.35 (20.05)	7.27 (23.82)	67.36 (17.22)	75.00 (16.61)	7.64 (18.26)	72.02 (20.88)	75.00 (15.81)	2.98 (26.49)
Point to start, average deviation	22.04 (13.61)	23.71 (21.60)	1.67 (19.23)	32.77 (39.31)	33.35 (38.88)	0.58 (23.90)	24.64 (33.84)	24.76 (14.23)	0.13 (37.56)
Point to end, average deviation	28.44 (14.59)	34.78 (26.83)	6.34 (29.19)	22.42 (9.28)	35.11 (25.02)	12.69 (22.25)	27.17 (12.38)	35.63 (16.14)	8.46 (14.77)
Location on map, average pixels	128.40 (68.54)	121.26 (83.95)	−7.14 (85.90)	110.91 (79.53)	80.19 (57.32)	−30.72 (72.83)	114.70 (56.18)	110.72 (63.06)	−3.98 (75.01)
**First-Person Perspective**								
Route sequence, % correct	59.59 (28.33)	61.34 (28.58)	1.74 (32.23)	64.58 (36.19)	75.00 (26.78)	10.42 (49.31)	60.71 (25.40)	72.62 (24.24)	11.90 (26.36)
Route continuation, % correct	70.93 (18.44)	70.93 (18.84)	0.00 (22.66)	74.31 (18.92)	76.39 (22.23)	2.08 (21.54)	65.48 (19.73)	66.67 (17.38)	1.19 (27.64)
Distance estimation, % correct	63.66 (21.27)	62.50 (17.25)	−1.16 (26.70)	70.83 (16.04)	64.58 (20.22)	−6.25 (26.52)	68.45 (21.51)	63.10 (19.56)	−5.36 (28.66)
Point to start, average deviation	47.64 (19.67)	47.63 (20.44)	0.00 (27.94)	48.85 (28.14)	44.74 (24.00)	−4.11 (17.71)	47.75 (14.05)	42.31 (17.47)	−5.43 (23.74)
Point to end, average deviation	48.07 (22.53)	60.75 (29.84)	12.68 (31.99)	46.92 (21.64)	48.14 (21.67)	1.22 (25.68)	58.20 (24.10)	61.19 (23.71)	3.00 (32.56)
Location on map, average pixels	139.96 (73.62)	163.24 (89.51)	23.28 (87.74)	135.69 (69.65)	139.62 (72.10)	3.93 (56.38)	149.54 (69.87)	152.72 (68.59)	3.18 (68.65)

### Subjective Navigation Ability

MANOVAs were performed to test the hypothesis that navigation training leads to an increased rating of subjective navigation ability on the “Navigation and Orientation” and “Distance Estimation” scales and decreased score on the “Spatial Anxiety” subscale, in the experimental groups compared to the control group (Table 4). No main effect of condition on difference scores was found, *F*_(6,148)_ = 1.29, *p* > 0.05; Wilk’s *Λ* = 0.90, partial η^2^ = 0.05.

**Table 4:** Scores on the Wayfinding Questionnaire pre- and post-training.

	Control			Egocentric training			Allocentric training		
Wayfinding questionnaire subscales	T1	T2	ΔT1.2	T1	T2	ΔT1.2	T1	T2	ΔT1.2
Navigation and orientation	50.16 (9.96)	50.47 (9.79)	0.30 (3.91)	49.44 (8.54)	50.56 (7.37)	1.11 (3.80)	49.62 (9.35)	48.90 (10.11)	−0.71 (4.79)
Distance estimation	11.44 (3.19)	11.42 (3.49)	−0.02 (2.23)	11.00 (2.83)	10.61 (2.91)	−0.39 (1.24)	11.19 (3.71)	11.57 (3.80)	0.38 (1.99)
Spatial anxiety*	21.42 (8.34)	22.19 (8.25)	0.77 (3.89)	21.78 (5.48)	22.06 (6.91)	0.28 (3.77)	24.24 (8.19)	23.76 (8.70)	−0.48 (3.93)

Displayed values are mean scores, and standard deviations in brackets. *A higher score represents a higher level of spatial anxiety.

### Interaction Between Strategic Preference, Preference Shifts, and Navigation Abilities

MANOVAs were performed to explore the relation between strategic preferences at T1 an objective and self-reported navigational abilities. Performance on egocentric and allocentric spatial tasks did not differ between participants with allocentric, egocentric or mixed strategic preference, *F*_(8,148)_ = 1.51, *p* > 0.05; Wilk’s *Λ* = 0.85, partial η^2^ = 0.08. Similarly, self-reported navigation abilities did not differ between subjects with different strategic preferences *F*_(6,152)_ = 0.26, *p* > 0.05; Wilk’s *Λ* = 0.98, partial η^2^ = 0.01.

To explore differences in egocentric and allocentric spatial abilities between participants that shifted strategy after the intervention and those who maintained the same strategic preference, a MANOVA was performed. Performance on egocentric and allocentric tasks did not differ between strategy shifters and non-shifters, *F*_(4,75)_ = 0.82, *p* > 0.05; Wilk’s *Λ* = 0.96, partial η^2^ = 0.04. Similarly, self-reported navigation abilities did not differ between strategy shifters and non-shifters, *F*_(3,77)_ = 0.26, *p* > 0.05; Wilk’s *Λ* = 0.99, partial η^2^ = 0.01.

Binary logistic regression analysis was performed to determine whether objective navigation abilities would predict shifts in strategic preference. Shifts in strategic preference were not predicted by objective navigation abilities, $\chi_{\left(4\right)}^{2}$ = 2.2, *p* = 0.69, or self-reported navigation abilities $\chi_{\left(3\right)}^{2}$ = 0.54, *p* = 0.91 at T1.

## Discussion

There is a strong need to develop rehabilitation programs for acquired brain injury patients with navigation impairments. A core approach to cognitive rehabilitation is the application of compensatory strategies. In the current study, we assessed the effectiveness of a home-based rehabilitation software designed to train and develop alternative navigation strategies in healthy participants.

The current study shows that strategic navigation preference can be influenced by using a navigation training program. A large portion of the participants that received the egocentric navigation training shifted from an allocentric or mixed navigation strategy preference before training, to an egocentric navigation strategy preference after training. This shift in strategic preference was the result of the training intervention as the proportion of shifters observed in the control group was significantly lower. Exploration of the individual characteristics of participants indicated that strategy shift was not predicted by a demographic factor such as gender, education or gaming experience. Furthermore, objective and self-reported navigation abilities did not predict strategic preference shifts. While an earlier study has shown that navigation strategy can be influenced by the use of intensive therapy sessions (Claessen et al., 2016a), these findings provide support for the hypothesis that strategy training can be achieved by the use of a standardized home-training program in combination with psychoeducation.

Important to note, however, is that the increase in strategy shifts was only demonstrated for the egocentric strategy training program. Participants who engaged in the allocentric training did not change strategy more often than the control groups. These results suggest that the current home training program was ineffective in inducing an allocentric navigation strategy. There are several factors that might explain why the allocentric training seemed to be ineffective in altering strategy preference.

First, the training time was significantly higher in the egocentric training condition compared to the allocentric training condition. This difference was the result of inherent differences between the training modules that were used in both programs. The duration of the allocentric modules was mostly dependent on the skill of the participant, as the goal of the modules was to find the shortest path to a location. Conversely, the turn sequence and landmark-action modules in the egocentric training required participants to traverse lengthy routes through an environment regardless of a participant’s skill level. While a higher training time was observed in the egocentric training condition, a significantly higher number of trials were attempted and completed in the allocentric training. Exploratory analysis revealed that within the experimental groups, training time did not predict the strategic preference shift. Conversely, a lower number of trials completed predicted a higher chance of preference shifts. Clearly, exposure time and the number of exercises were not the most prominent factors that predict training success. Rather, the content and presentation of the training exercises in the allocentric training modules should be improved. A small number of lengthy trials seemed to be preferable over many short trials for the development of navigation strategies.

A second explanation for the lack of strategy shifts observed after allocentric training regards the difficulty of switching between allocentric and egocentric reference frames during navigation. Egocentric navigation entails a focus on landmark-response associations, sequences, and spatial updating rather than forming relational representations (Bullens et al., 2010). Conversely, the formation and utilization of map-like representation of space are central to allocentric navigation. Constructing such allocentric representations is cognitively demanding (Wen et al., 2011; Nemmi et al., 2017; Ruggiero et al., 2018). Furthermore, a considerable processing cost is involved in switching between egocentric and allocentric reference frames (Lee and Tversky, 2001). As such, shifting from an allocentric to an egocentric navigation strategy reflects a shift towards a strategy that is cognitively less demanding, whereas a switch from an egocentric to an allocentric navigation strategy, can be regarded as a switch to a more demanding strategy. The environment used to assess the navigation strategy in this study was developed to facilitate both allocentric and egocentric strategies (Iglói et al., 2009). It is, therefore, possible, that participants who received the allocentric training, were not prompted by the environment to adopt the trained strategy and instead reverted to their default strategy.

Related this explanation are the results reported by Pazzaglia and Taylor (2007), who examined the cognitive style of spatial processing in participants with high and low survey abilities. In this study, participants with high survey abilities were less depended on learning perspective and were able to shift more efficiently from one representation to another compared to participants with low survey abilities. A similar effect was found when regarding the participants with a naïve allocentric preference as the high survey participants, as participants with an allocentric strategic preference were more responsive to the training. One important difference with this study however, is that naïve strategic preference did not correspond performance in objective navigation tasks in this study.

In addition to a shift in strategic navigation preference, we expected that exposure to the training programs would lead to an increase in objective navigation ability and self-reported navigation ability. Contrary to expectations, no effect of the training was found on both objective and subjective navigation ability. This result indicates that the strategy training did not strengthen specific navigational abilities, but rather, affected meta-cognition and behavioral selection. Additionally, we did not find differences in objective navigational abilities between the groups before the training. Preferred strategy during the pre-training session, did not correspond to higher performance on allocentric or egocentric objective navigation abilities. This finding supports a study that has shown that strategic navigation preference does not correspond to navigation ability (Prestopnik and Roskos-Ewoldsen, 2000). The relation between strategy preference and navigation skills has yet to be studied thoroughly, but might be of particular importance to the rehabilitation of navigation impairments. It appears that someone’s preferred navigation strategy is not grounded in their spatial strengths and weaknesses. When developing compensatory strategy therapies for navigation impaired patients, care should be taken to make patients aware of their strengths and focus their efforts to maximizing the use strategies that utilize these abilities.

An important distinction between this study and the intended clinical application should be noted. In order to assess whether strategy use can be changed, participants were trained to adopt a navigation strategy that was contrary to their initial preferences. Patients however, will be trained to focus on and expand upon their intact navigation abilities. Ineffective strategies and abilities will be recognized and discouraged, while effective a strategies and abilities will be expanded upon. As the training is tailored to their strengths, rather than to their weakness, we expect that it will be easier for patients to utilize the training and transfer this information to real life situations.

Furthermore, the rehabilitation training that was investigated here focused on promoting the use of allocentric and egocentric navigation strategies. Both strategies rely on the use of landmarks. There have been rapports of patients with specific impairments in landmark recognition, encoding and processing (Rainville et al., 2005). Therefore, future therapies should be developed that train navigation strategies that include a minimal focus on landmarks.

Using the current iteration of the navigation training, participants with an egocentric navigation strategy preference did not adopt an allocentric navigation strategies. While it might not be possible to train allocentric navigation strategies, we expect that improvements to the training program will lead to training success. Based on the findings of this experiment, we propose to following improvements. First, fewer but lengthier training modules in the allocentric training. One explanation for the training success of the egocentric strategy training is the longer training time compared to the allocentric training. Second, as the “distal landmarks” and “local landmarks” place learning modules might have been too similar in terms of what navigation techniques were taught. A larger variety of training modules in the allocentric might be beneficial to strategy development. Third, an extended discussion of an individual’s strengths and weaknesses during the psychoeducation phase of the training. The results suggest that people display navigation strategies that are not necessarily in line with their spatial abilities. Making people aware of their strengths and weaknesses might lead to higher adherence to beneficial navigation strategies. More research should be performed to determine whether a change towards an allocentric strategy preferences can be achieved when these novel features are implemented.

Over the past years, there has been a growth in software applications that combine game-like features with health related goals such as diagnosis of cognitive impairments. Spatial cognition in particular, lends itself well to serious-gaming adaptations as illustrated by applications such as “Sea Hero Quest” (Coutrot et al., 2018), “Navigeren kun je leren” and “Squirrel away” (Prpic et al., 2019). While substantial progress is being made in regards to the diagnosis of spatial impairments using these tools, the validity of treatment applications has yet to be explored. In context of this emerging field, the current study provides the encouraging results for a compensatory approach to the rehabilitation of navigation impairments using a game-like application.

In conclusion, we have developed a home-based rehabilitation training designed to treat navigation impairments that are often reported in acquired brain injury patients. A key assumption of this training is that strategic navigation preferences can be influenced by using a training. This study demonstrates that strategic navigation preference can indeed be influenced in healthy participants. Allocentric navigators could be trained to adopt an egocentric strategic preference. The current version of the training, did not induce a change in strategic preference in egocentric navigators. This may be due to factors inherent to the allocentric training such as its focus on multiple short exercises or a lack of diversity between exercises. Alternatively, switching from an egocentric to an allocentric navigation strategy, requires a switch towards a strategy that is cognitively more demanding. Egocentric navigators might not have been prompted to rely on the trained strategy in an environment, which was ambiguous regarding navigation strategies. Future research should be conducted to optimize the training for acquired brain injury patients with navigation impairments. The feasibility and effectiveness of the current approach should next be assessed in a patient population.

## Data Availability Statement

The datasets generated for this study are available on request to the corresponding author.

## Ethics Statement

The studies involving human participants were reviewed and approved by CEP FSW LEIDEN. The patients/participants provided their written informed consent to participate in this study.

## Author Contributions

MK, AE, JV-M, and IH conceptualized, prepared the original draft and wrote the manuscript. MK and IH contributed to the methodology. MK was responsible for the formal analysis and investigation. IH was responsible for the funding acquisition. AE, JV-M, and IH supervised the study.

## Conflict of Interest

The authors declare that the research was conducted in the absence of any commercial or financial relationships that could be construed as a potential conflict of interest.

## Footnotes

^1^Fisher’s exact test did not reveal a significant difference in the proportion of strategy changers between the “control” and (14.3%) “control + psycho-education” conditions (23.8%; *p* = 0.69; FET). In the remainder of the analysis, the control groups were combined to enhance the power of the analyses.
