# Supplementary material for: The Effectiveness of Home-Based Training Software Designed to Influence Strategic Navigation Preferences in Healthy Subjects
Source: Front Hum Neurosci. 2020 Mar 17;14:76. doi: 10.3389/fnhum.2020.00076 (PMC7092635; doi:10.3389/fnhum.2020.00076)
Supplement: Supplementary file 1 [file Data_Sheet_1.docx]

Supplementary Materials

The effectiveness of home-based training software designed to influence strategic navigation preferences in healthy subjects

M.N.A. van der Kuil, A.W.M. Evers, J.M.A. Visser-Meily & I.J.M. van der Ham

# Home-based Navigation training

##### Egocentric Training

The egocentric training was composed of the modules: “landmark-action association”, “turn-sequence” and “egocentric updating”.

In the “***landmark-action association***” module, a virtual environment was constructed consisting of an array of 3-way intersection points connected by corridors. The corridors connected to the intersection points formed a 120 degree angle. At each junction point, one corridor served as entry point, one corridor lead to a dead end and one corridor lead to either the next junction point or to the exit (Supplementary Figure 1). A square landmark was placed in between the two response corridor, facing the entrance corridor. A mist was present in the environment, obscuring the rooms at the other end of the corridors.

In each trial, an environment was generated using a semi-randomized procedure. This determined the order the of intersection points along the route (e.g. left, right, right, left). Landmarks were randomly selected from a database of 46 images (black and white symbols).


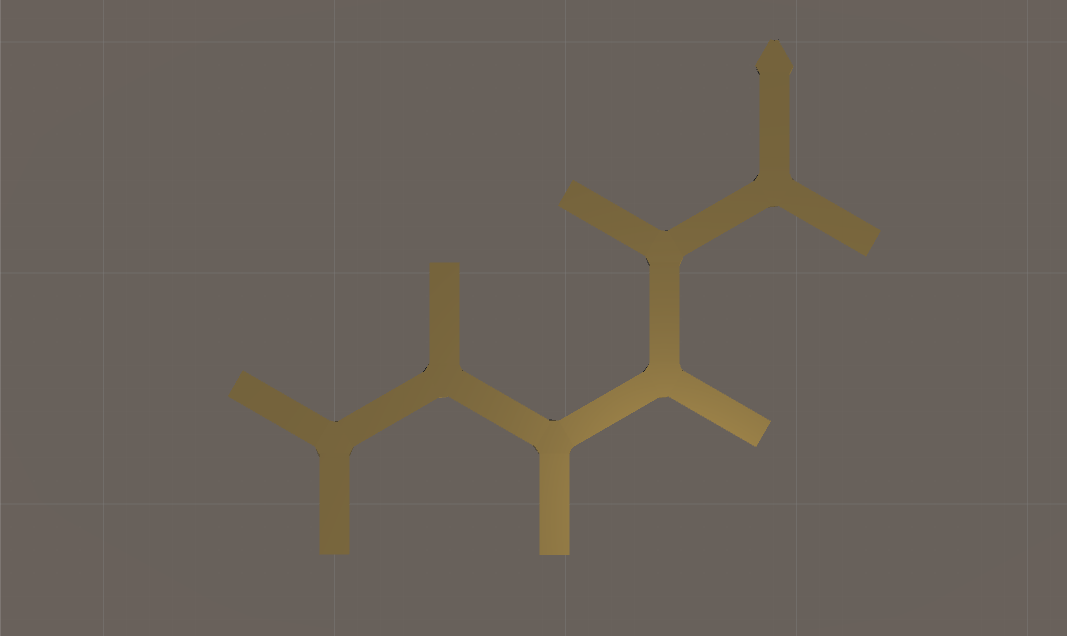


**Supplementary Figure 1.** Overview of an environment as constructed through a randomized procedure in the landmark-action association and turn sequence modules.

A demo route through the maze was shown. Participants were instructed to remember what action was taken at each landmark (Supplementary Figure 2). Afterwards, participants were placed in the maze and had to find their way to the ending location. When visiting a dead end, a point was subtracted, when entering the correct corridor, a point was earned.

The difficulty of a level was determined by how many intersection points were present in the environment: difficulty block 1 contained 3 levels consisting of 2, 3 and 4 intersection points, difficulty block 2 contained 3 levels consisting of 4, 5, 6 intersection points, etc.


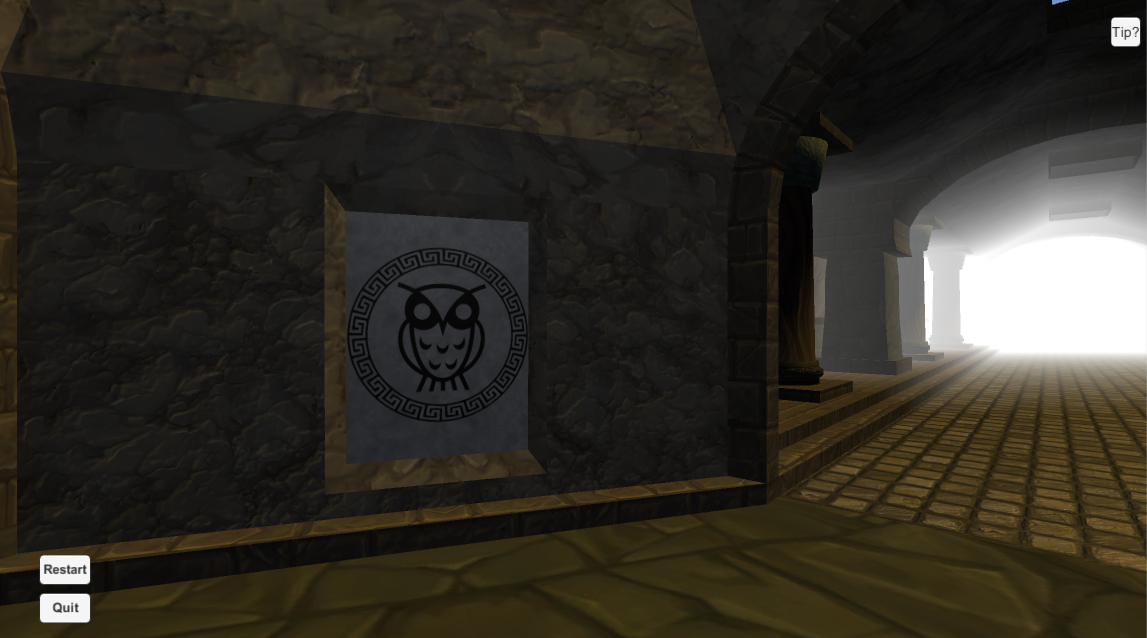


**Supplementary Figure 2**. Landmarks are presented at intersection points in the landmark-action association module.

The “***turn-sequence***” module, was similar to the “*landmark-action association*” module with two important exemptions. First, no landmarks were present in the environment (Supplementary Figure 3). Second, the participants were instructed to remember the order of turns taken as shown in the demo video. Thus, participants could only find their way to the ending location by encoding and reproducing a egocentric turn sequence.

**
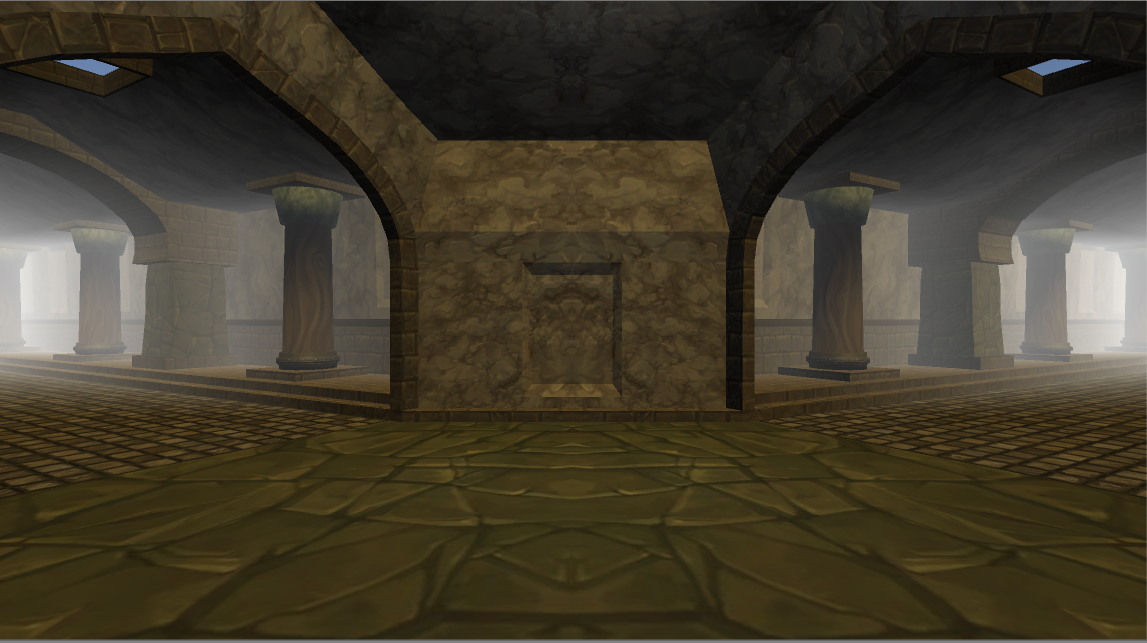
**

**Supplementary Figure 3**. An intersection as seen in the turn sequence module.

In the “***egocentric updating***” module, a virtual environment was constructed consisting ending and starting room connected by a single corridor (Supplementary Figure 4). This corridor was composed of three types of interconnected sections: 90 degree left turns, 90 degree right turns and strait sections. Ten measurement locations were present in each environments.

In each trail, participants had to remember the location of the starting room, while traveling through the corridor to the ending room. The participants were instructed to maintain their sense of direction, by imagining a compass always pointing to the starting location. When a participants arrived at a measuring point, an arrow would be shown in front of the camera pointing forward (Supplementary Figure 5). Participants were tasked to point towards the starting location by rotating the camera. After pointing, the walls in the environment became transparent (Supplementary Figure 6). This allowed participants to observe their pointing deviation and recalibrate their orientation. Points were earned depending on the pointing deviation: a deviation between 0 and 30 degrees resulted in 2 coins earned, a deviation between 30 and 60 degrees resulted in 1 coin earned. A deviation greater than 60 degrees resulted in 0 coins earned. After recalibrating the wall turn opaque and participants proceeded further along the corridor.


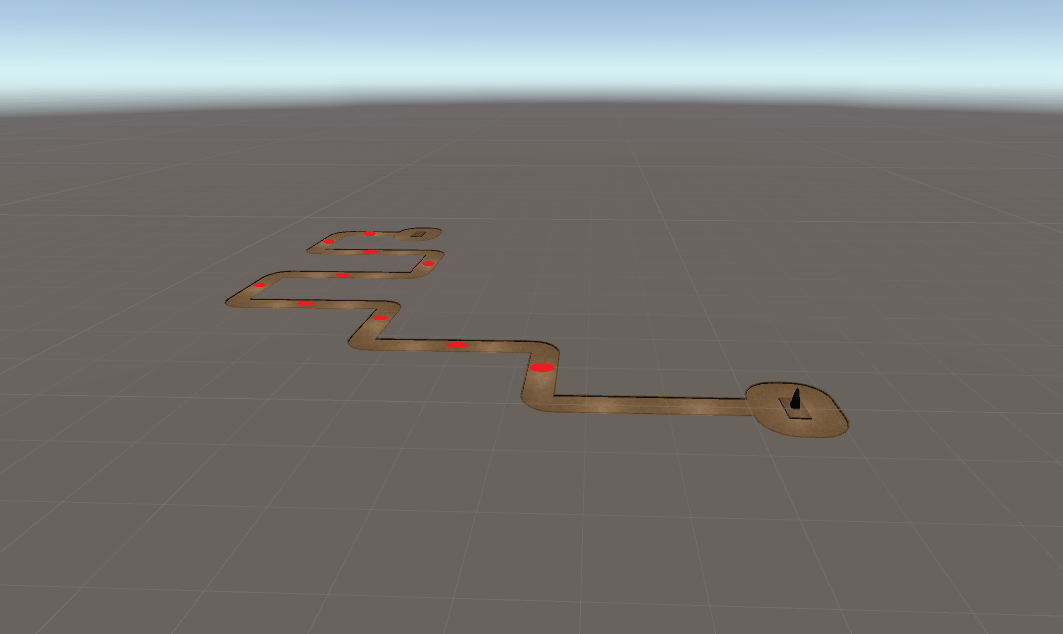


**Supplementary Figure 4.** The layout of an environment constructed in the egocentric updating module. The black pyramid in the starting room is the target of the pointing task. Red dots indicate measuring points, these were not visible to the player while traversing the environment.


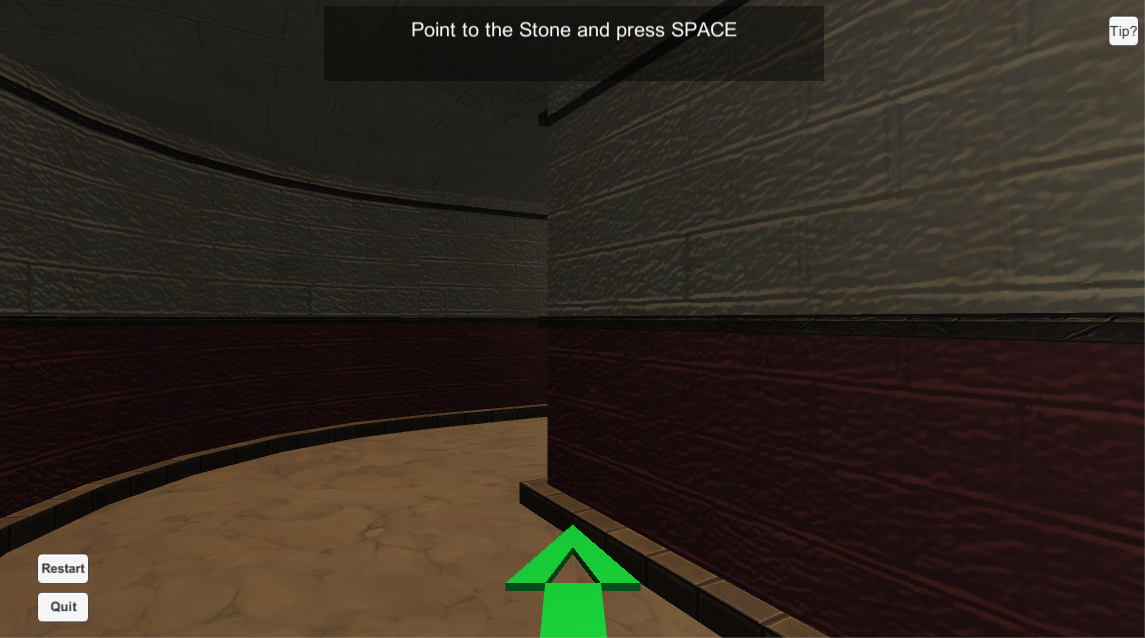


**Supplementary Figure 5.** The measuring phase in the egocentric updating module requires payers to orient the arrow to towards the starting room.

**
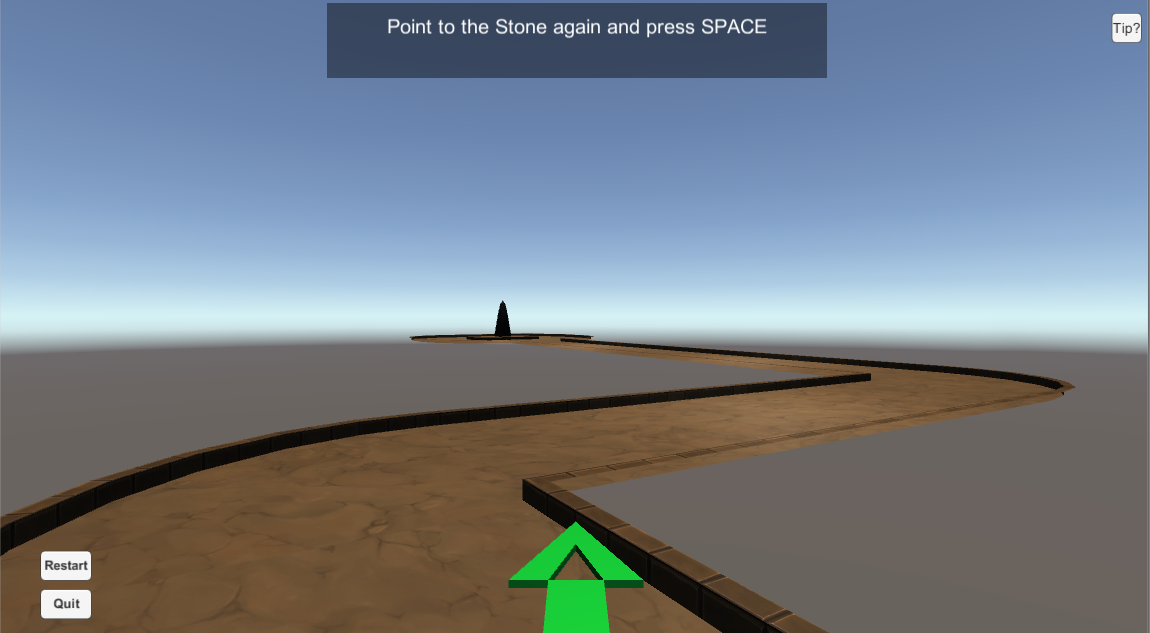
**

**Supplementary Figure 6.** Feedback in the measuring phase, after pointing the walls of the environment become transparent and navigators can reorient themselves towards the starting location.

The difficulty of a level was determined by the amount of sections that composed the corridors, and thus the distance traversed between measuring points: Difficulty block 1 contained 3 environments with 10 sections (each with a measuring point), or 20 sections (a measuring point alternately placed between non-measuring point sections). Difficulty block 2 contained 3 environments with 20 sections or 30 sections (a measuring point alternately placed between 2 non-measuring point sections), etc.

##### Allocentric Training

The allocentric training was composed of the modules: “place-finding: distal landmarks”, “place-finding: local landmarks” and “map-use”.

In the “***place finding: distal landmark***” module, a virtual environment was constructed consisting of circular platform surrounded by 1 to 4 landmarks that were placed outside of the platform in the north, south, east or west direction. A start location and target location was present in the environment. In each trial, an environment was generated using a randomization procedure. The procedure determined the identity and locations of the landmarks. Furthermore, the starting and target locations were selected from a list of 48 coordinate combination.

At the start of a trail, participants were presented with a 2D map of the environment (Supplementary Figure 7). The start location was not visible on the map. The target location was indicated with a red dot. After studying the map, participants were placed on the starting location. The target location was not visible in this first-person perspective. Participants were instructed to use the distal landmarks and their relative position on the platform to find the shortest path to the target location (Supplementary Figure 8). A ‘step counter’ bar was present on the top of the screen. Traversing distance cause the bar to decrease. If participants traveled less than 2 time the minimal 2 points were earned. If participants travelled 2 to 4 times the minimal distance they would earn 1 point. If participants travelled more than 4 times the minimal distance to reach the target location, no points were earned.

**
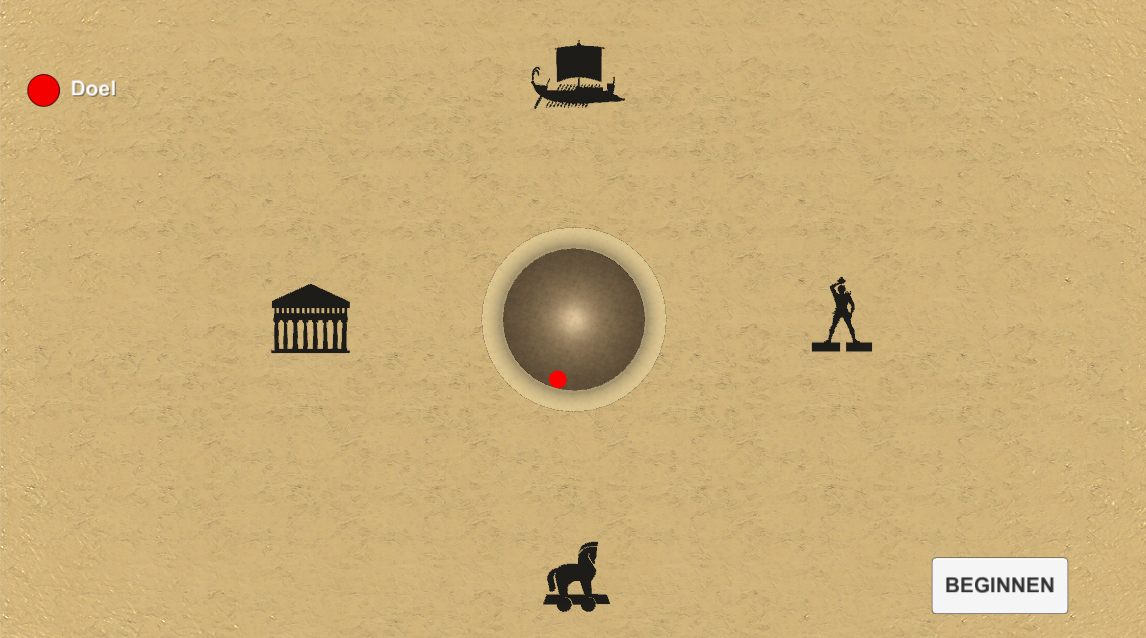
**

**Supplementary Figure 7.** A map is presented at the start of the trial that contains the target location (red dot) and the distal landmarks (black figures).


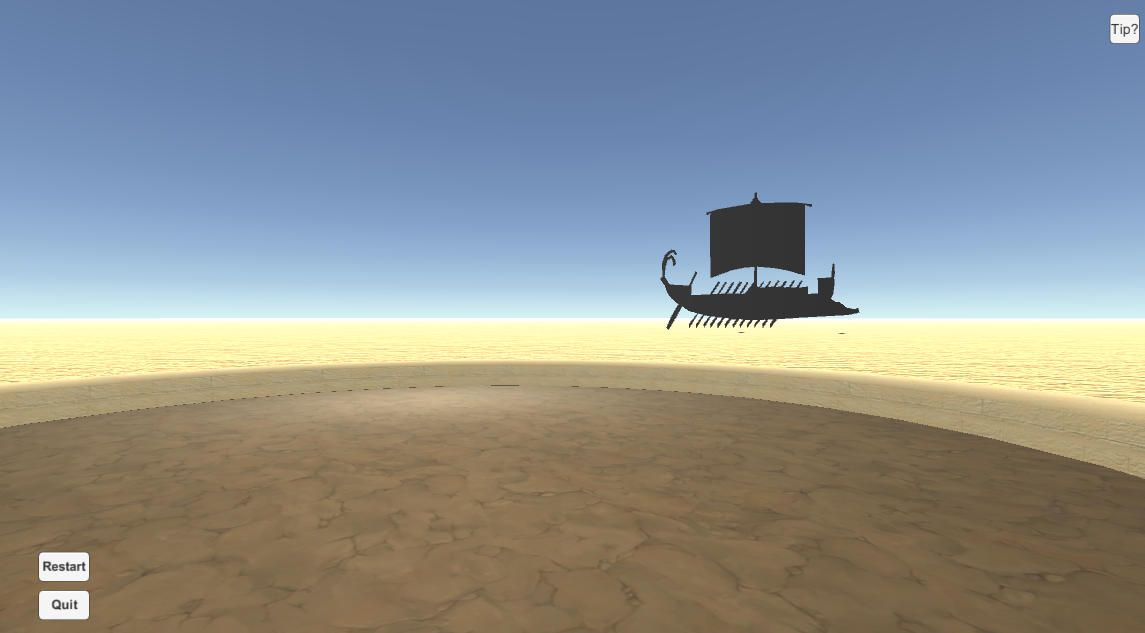


**Supplementary Figure 8.** Players use distal landmarks to find the target location.

The difficulty of a level was determined by the amount on distal landmarks present in the environment and the size of the circular platform itself. Difficulty block 1 contained 4 landmarks. Within a each difficulty block, participants would perform 3 trails in small, medium and large platforms.

The ***“place finding: local landmarks”*** module, was similar to the “*landmark-action association*” module with one important exemption. No distal landmarks were present in this module. Instead, 3 local landmarks (pillars) were placed inside the circular platform (Supplementary Figure 9). Participants were instructed to find the shortest path the target by using the configuration of the 3 pillars in relation to their own location and the location of the target.

The difficulty of a level was determined by the placement of the target location in relation to the local landmarks. In the first difficulty block, the target location was always placed and the foot of a pillar. In the second difficulty block, the target was placed on a ‘line’ between two pillars. In the third difficulty, the target was placed inside the ‘triangle area’ formed by configuration the three pillars. In the fourth difficulty block, the target was placed outside the ‘triangle area’ formed by the configuration of the three pillars.


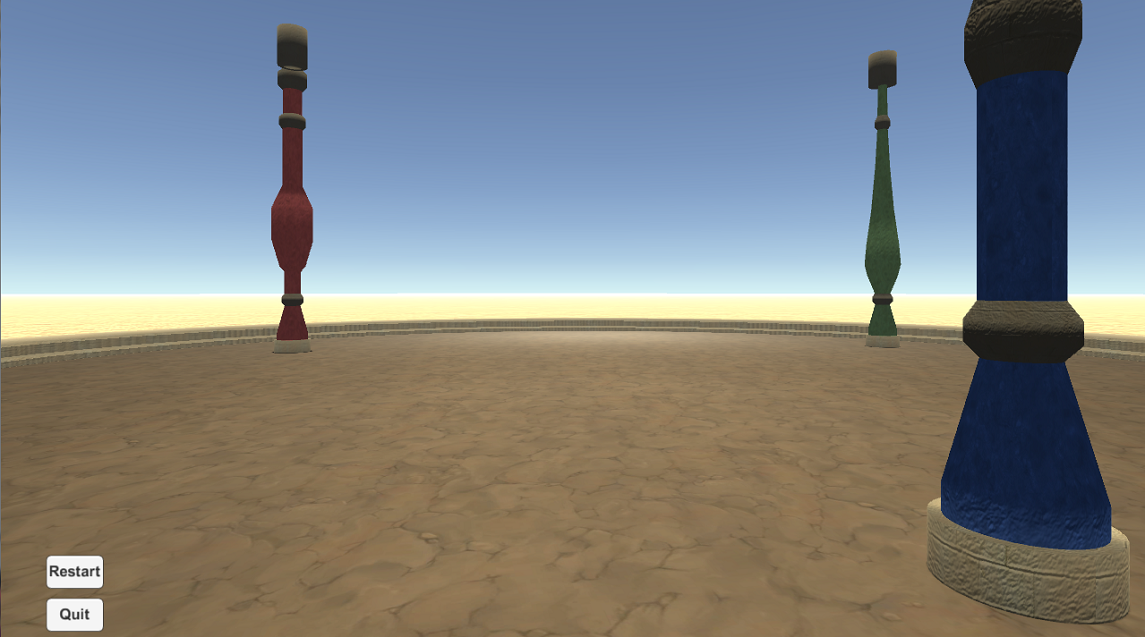


**Supplementary Figure 9.** Players use local landmarks to find the target location.

In the “***map-use***” module, a virtual environment was generated consisting of a variable number of square rooms placed in a grid formation. Each room had 4 corridors. Corridors connected to the adjacent rooms or to dead ends (in rooms in the outer layer of the environment). A randomization procedure filled each room with landmarks derived from a database of 46 images (black and white symbols), ‘red’ landmarks, a start and an end location. The starting and ending rooms were randomly determined but a set travel distance between these rooms was always maintained.

Depending on the trial condition, participants had access to ‘dynamic’, ‘static’ or ‘temporary’ map information. The map was a 2D overview of the environment depicting the rooms, landmarks and ending location (red dot). In the ‘dynamic’ and ‘static’ conditions participants were presented with a split-screen. In the top screen, a first-person perspective of the environment was shown. In the bottom screen, a map of the environment was shown. In the ‘dynamic’ condition, a participant’s current location was updated and shown on the map (blue dot). In the ‘static’ condition, the starting location was shown on the map (blue dot), but the current location was not shown (Supplementary Figure 10). In the ‘temporary’ map condition, a map of the environment was only show prior to navigating in the environment. The map could be studied as long as required and disappeared after starting the trial, leaving only the first person perspective.

Participants were instructed to use the map to find the shortest route to the ending room and avoid visiting the rooms containing a ‘red’ landmarks. Participants started with a number of points. Visiting the room resulted in a point lost. Visiting a room with a ‘red’ landmark lead to a loss of 2 points.

Difficulty In the “*map-use*” module was determined by the size of the environment. Difficulty block 1 contained an environment consisting of 9 (3x3) rooms. Difficulty block 2 contained an environment consisting of 16 (4x4) rooms. During each block participants completed a ‘dynamic’, ‘static’ and ‘temporary’ map condition in 3 randomly generated environments.


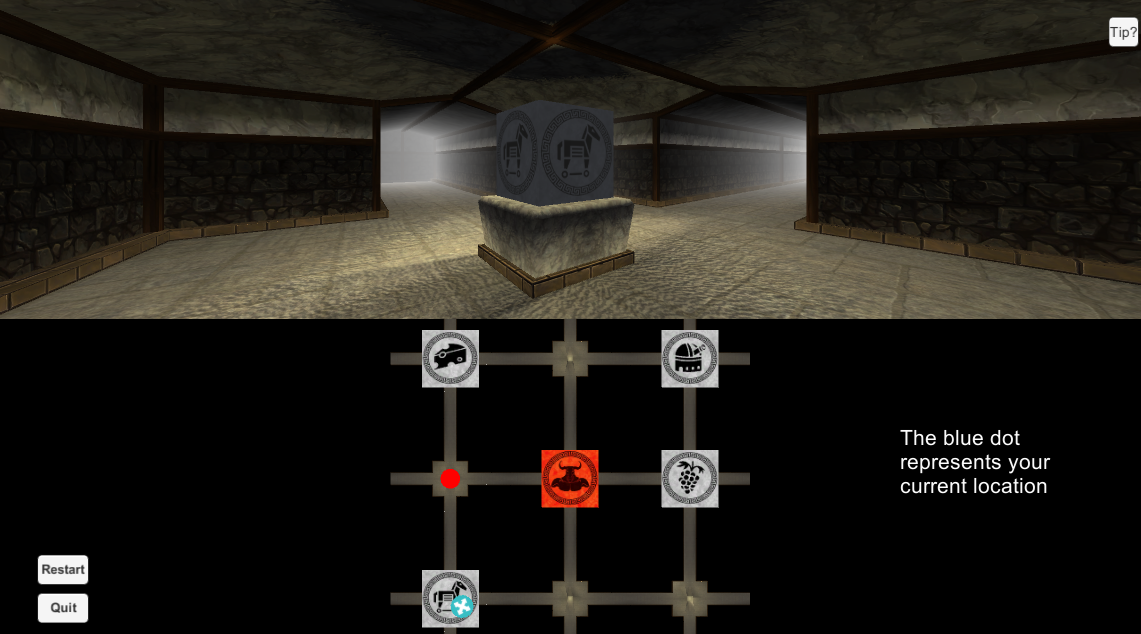


**Supplementary Figure 10.** Players can use the map in the bottom screen to orient themselves in the environment. The map shows their current location (blue dot), the goal location (red dot), the landmarks and the red landmarks.
